# Frequency of neuropathic pain and its effects on rehabilitation outcomes, balance function and quality of life among people with traumatic spinal cord injury

**DOI:** 10.12669/pjms.38.4.4681

**Published:** 2022

**Authors:** Muhammad Idrees Khan, Aatik Arsh, Ikram Ali, Aman Khan Afridi

**Affiliations:** 1 Muhammad Idrees Khan, DPT, MSPT. Lecturer, Department of Health Sciences, City University Peshawar, Pakistan; 2 Aatik Arsh, DPT, MSPT. Assistant Professor, Institute of Physical Medicine and Rehabilitation, Khyber Medical University Peshawar, Pakistan; 3 Ikram Ali, BSPT, TDPT, MSPT. Assistant Professor, Institute of Physical Medicine and Rehabilitation, Khyber Medical University Peshawar, Pakistan; 4 Aman Khan Afridi, DPT, MSPT. Lecturer, Department of Health Sciences, City University Peshawar, Pakistan

**Keywords:** Neuropathic Pain, Rehabilitation outcomes, Balance Function, Quality of Life, Traumatic Spinal cord injury

## Abstract

**Background & Objectives:**

Traumatic Spinal cord injury (SCI) is a devastating condition that results in life long disability. Impairments associated with traumatic SCI such as sensory, motor, and autonomic dysfunctions lead to an array of secondary SCI-specific complications. Neuropathic pain is one of the most common medical complications of traumatic SCI which significantly affects motor function and activities of daily living (ADL) in people with traumatic SCI. Neuropathic pain is one of the main factors for dependency, decreased quality of life (QOL), poor rehabilitation outcomes, and depression in traumatic SCI individuals. The main aim of the current study was to determine the frequency of neuropathic pain and its effects on rehabilitation outcomes, balance function, and QOL in people with traumatic SCI.

**Methods:**

A cross-sectional survey was carried out at PCP from March to August 2020. Overall, 123 participants were added to the study using a non-probability convenience sampling technique. Information was collected using an adapted, validated questionnaire. Both male and female traumatic SCI patients with age between 18-60 years who received at least two weeks of rehabilitation, 42 days after diagnosis of traumatic SCI were included in current study while patients with Acute SCI, SCI patients with any other condition which can affect neuropathic pain such as traumatic brain injury, diabetic neuropathy, amputation, etc. and progressive neurological diseases such as multiple sclerosis and Guillain barre syndromes were excluded. Patients who have received at least two weeks of rehabilitation, 42 days after diagnosis of traumatic SCI. Patients with traumatic SCI.

**Results:**

Overall, 123 traumatic SCI patients were included in the study. The majority of the (n=101, 82%) participants were male and 83 (67.5%) were from urban areas. Eighty-Seven (70.73%) participants had neuropathic pain. Neuropathic pain was significantly associated (P-value <0.005) with rehabilitation outcomes, balance function, and quality of life.

**Conclusion:**

It can be concluded that more than two-third of SCI patients suffer from neuropathic pain. Moreover, neuropathic pain is significantly associated with rehabilitation outcomes, balance function, and quality of life.

## INTRODUCTION

Traumatic SCI is a devastating condition that results in lifelong disability.^1^ Impairments associated with traumatic SCI such as sensory, motor, and autonomic dysfunctions lead to an array of secondary SCI-specific complications.^2^ Neuropathic pain is one of the most common medical complications of traumatic SCI which significantly affects motor function and ADL in people with traumatic SCI.^3^ Neuropathic pain increases patient dependency, decreases patient’s QOL, poor rehabilitation outcomes, and promotes psychological problems in traumatic SCI individuals.^4^ Globally, 27 to 94% of traumatic SCI patients suffer from neuropathic pain.^4^ In Pakistan, the reported prevalence of neuropathic pain ranges from 17 to 57.5%.^5^ With the passage of time and effective measures, the number of traumatic SCI individuals may decrease.^6^ There is one patient out of tens of patients of traumatic SCI patients having neuropathic pain. If this ratio continues, the number is expected to be one out of five patients and so on one out of three in 2051.^7^

The symptoms of neuropathic pain generally persist for long durations.^8^ The neuropathic pain symptoms often do not respond to pharmacological and electrotherapeutic modalities.^9^ Previous studies have reported that neuropathic pain increases the suffering of traumatic SCI individuals.^10^ With the increase in symptoms of neuropathic pain, the physical function of the human body decline, and the individuals suffers from various health problems.^11^ Traumatic SCI patients with neuropathic pain are confronting a wide range of psychological problems which in turn reduces the effectiveness of rehabilitation interventions.^12^ These patients had poor compliance with physical and occupational therapy and they have a longer length of stay at rehabilitation centers.^13^ Due to all these reasons, the treatment of the traumatic SCI patients with neuropathic pain is more expensive as compared to the traumatic SCI patients without neuropathic pain.^14^

Neuropathic pain is a disabling condition that restricts patients from performing ADLs and affects the patient’s QOL.^15^ Limited physical activity causes many health-related problems in traumatic SCI patients such as the immune system becomes weak which may also lead to many diseases.^16^ The majority of the diseases in traumatic SCI patients are due to the change in lifestyle, emotional impact, social circumstances, nutrition, and psychosocial health.^17^

## METHODS

A cross-sectional study was conducted in PCP, Khyber Pakhtunkhwa, Pakistan. This hospital is a semi-government hospital located at the center of Khyber Pakhtunkhwa. This hospital covers 80% of the SCI population of the KPK province and considers a hub for all rehabilitation sector hospitals. Participants who fulfilled the inclusion criteria have been selected from different departments of that hospital. The study was conducted in six months (March 1^st^, 2020 to August 30^th^, 2020).

The study was conducted after the approval of ASRB and Khyber Medical University’s ethical and research committee, letter no. DIR/KMU-EB/FA-000792 dated 5-6-2020. The approval for the data collection was granted on the basis of said ethical report by Chief Executive Officer of the Paraplegic Center Peshawar. Before data collection, the aims and objectives of the study were elaborated to the subjects and the concerns of the participants were cleared. After this, the participants were selected via non-probability convenience sampling technique for the current study. There were enough traumatic SCI patients in that hospital that were easily accessed through non-probability convenience sampling. Initially, the list of traumatic spinal cord injury patients in the hospital was collected. After that 123 traumatic spinal cord injury patients who fulfilled the inclusion criteria were selected from different units of the hospital.

### Inclusion & Exclusion criteria:

The patient included were male and female traumatic SCI patients age 18-60 years, patients who received at least two weeks of rehabilitation, 42 days after diagnosis of traumatic SCI and Patients only having traumatic SCI. Similarly the excluded candidates for the study were Acute SCI patients, SCI patients with any other condition which can affect neuropathic pain such as traumatic brain injury, diabetic neuropathy, amputation, etc. and progressive neurological diseases such as multiple sclerosis and Guillain barre syndromes.

The data was collected using adapted questionnaires such as LANSS pain scale, SCIM, functional reach test, and WHO-QOL BREF among traumatic SCI patients. The questionnaire consisted of five sections. Section “A” covered demographic data. There were fifteen questions in section “A” regarding the age of the participants, gender, residence, marital status, occupation, height, weight, BMI, date Injury, cause of injury, radiological sign, spinal fixation, neurological Level, ASIA impairment scale and contact number.

The section “B” consisted of seven questions based on pain and sensory testing of traumatic SCI Patients. The first five questions were pain-related while the last two were symptoms related. The total score on the LANSS scale was 24. Those patients having pain score greater or equal to 12 were considered neuropathic pain while in patients having pain score less than 12 on the LANSS scale were considered non-neuropathic pain.

Section “C” consisted of two portions. The first portion was about the self-care of the patients have further four parts (1) Feeding (2) Bathing (3) Dressing (4) Grooming while the second portion was about the mobility of the patients. It had two parts. The first part was about indoor activity and other part was about outdoor activity.

Section “D” also consisted of two portions. The first portion was about the forward reach of both the right and left side of the patients while the second portion was about the lateral reach of the right and left side of the patients.

Section “E” was divided into four domains. These four domains included the physical health of the patients, psychological state of the patients, social relationship of the patients and the environment of the patients.

## RESULTS

Overall included participants in this study were 123 in which no participant quit the study. All the participants were traumatic SCI patients admitted in different units of PCP, Khyber Pakhtunkhwa. The maximum age of the participants was 60 years while the minimum age of the participants was 18 years. The mean age of the participants is 33.52 with a standard deviation of ±11.52. Analysis of the study showed that the majority (82.11%) of the study participants were male while (17.9%) were female.

Results declared that the majority (67.5%) of the study participants were reported from the urban area while (32.5%) SCI patients were noted from rural areas. The majority (30.09%) of the cause of injury of the participants was fall from a height, followed by road traffic accident (28.5%), fire arm injury (21.1%), hit by falling object (12.2%), coal mine accident (4.1), and fall on the ground (Table-I).

**Table-I:** Demographic profile of traumatic spinal cord injury patients (N=123).

Section	Features	Categories	Frequency	Percentage
A	Gender	Male	101	82.1%
		Female	22	17.9%
	Residence	Urban	83	67.5
		Rural	40	32.5
	Marital Status	Married	90	73.2
		Unmarried	33	26.8
	Occupation	Labor	46	37.4
		Housewife	19	15.4
		Driver	14	11.4
		Student	11	8.9
		Others	33	26.9
	BMI(kg/cm^2^)	BMI	25.65±4.71	-
	Cause of Injury	Fall From Height	38	30.9
		Road traffic accident	35	28.5
		Fire arm injury	26	21.1
		Hit by falling Object	15	12.2
		Others	9	7.4
	Radiological Sign	Cervical	20	16.3
		Thoracic	74	60.2
		Lumber	29	23.6
	Spinal Fixation	Yes	78	63.4
		No	45	36.6
	Neurological Injury Level	Cervical	20	16.3
		Thoracic	71	57.7
		Lumber	31	25.2
		Sacral	1	0.8
	Level Of Injury	Paraplegic	110	89.4
		Tetraplegic	13	10.6
	Asia Impairment Scale	Complete A	87	70.7
		Incomplete B	22	17.9
		Incomplete C	10	8.1
		Incomplete D	3	2.4
		Incomplete E	1	0.8
	Neuropathic Pain	Yes	87	70.73
		No	36	29.27

The majority (70.73%) of the traumatic SCI patients had neuropathic pain; followed by (29.27%) of traumatic SCI had no neuropathic pain (Table-II).

**Table-II:** Neuropathic pain in traumatic spinal cord injury patients (N=123).

Section	Neuropathic Pain	Number	Percentage
B	Patients with neuropathic pain	87	70.73%
	Patients without neuropathic pain	36	29.27%

The majority of the participants responded that their rehabilitation outcomes were affected by neuropathic pain because the study analysis shows that his p-value was below 0.05.

## DISCUSSION

The current study conducted shows that majority of the participants level injury is paraplegic and the ASIA impairment scale is complete. The basic purpose of the current study was to assess the frequency of neuropathic pain in traumatic SCI patients. The current study revealed that the majority n =87 (70.73%) patients had average signs and symptoms regarding neuropathic pain in traumatic SCI patients. As discussed earlier 70.73% of patients suffered from neuropathic pain in traumatic spinal cord injury while 29.27% had no pain. This rate was found to be higher at 17.73% than the previously reported study (53%) conducted by Burke D et al.^18^ Moreover, it also indicated a higher rate of neuropathic pain as compared to previously reported study conducted in the USA regarding the neuropathic pain in which prevalence ranged from 21% to 60% among the SCI population.^19^-^20^ In the same context, a study conducted in the UK to identify neuropathic pain showed that 70% of the SCI patients had signs and symptoms of neuropathic pain which is almost similar to the results of current study.^21^-^22^ Similarly, one of the study conducted in Australia reported higher percentage (77.3%) of the SCI patients as compared to the current study.^23^ A study conducted in the USA reported that neuropathic pain had a bad effect on rehabilitation outcomes and balance function in SCI patients which is in concurrence to the current study.^24^-^25^ It was observed druing the current study that neuropathic pain also effects the QOL of the SCI patients. It was also reported by De Oliveira B *et. al* that neuropathic pain effect QOL in SCI patients.^26^

**Table-III:** Neuropathic pain and its effect on rehabilitation outcomes, balance and QOL.

Section	Neuropathic pain and its effect on rehabilitation outcomes, balance	Neuropathic pain		P-Value*
		Yes (n=87)	No (n=36)	
C	Rehabilitation outcomes			
	Self –care	13.43±3.70	11.86±4.20	0.041
	Mobility	12.86±8.46	17.30±11.77	0.020
D	Balance function			
	FRT Right-Hand	17.44±7.93	21.20±7.93	0.016
	FRT Left-Hand	20.15±6.49	17.13±7.98	0.030
	LRT Right-Hand	21.47±8.0	18.13±7.89	0.037
	LRT Left-Hand	19.17±6.79	16.36±7.41	0.044
E	Quality of life			
	Physical Health	23.55±2.20	22.53±2.52	0.027
	Psychological	19.79±2.59	21.00±3.07	0.028
	Social Relationship	9.69±1.49	9.06±1.33	0.029
	Environmental	28.20±2.72	29.31±2.95	0.047

FRT: Functional reach test, LRT: lateral reach test,

* paired sample T test

### Limitation of the study:

Traumatic SCI patients from paraplegic center Peshawar were included in the study, nearly half of the traumatic SCI population admitted in tertiary care hospitals and private hospitals. It would be good if data were collected from traumatic SCI patients admitted in tertiary care hospitals.Time constraint is another limitation of the study.

## CONCLUSION

The research findings showed that neuropathic pain is one of the main issue among traumatic SCI patients.It can effect the rehabilitation outcomes, balance function and quality of life which can ultimately effect the general health status of such patients. Proper management and prognosis of neuropathic pain is important to improve the individual’s quality of life.This study will help in the management of effective therapeutic options by understanding the relevance of neuropathic pain among these patients.

### Authors’ Contribution:

**MIK:** Concept and study design, literature search and literature review,acquisition of data,drafting the manuscript,final approval of the version to be published. He is responsible for the accuracy or intergerity of the work. **AA:** Overall supervision,drafting the manuscript,analysis and interpretation of data. **IA:** Literature search and literature review,critical revision. **AKF:**Concept and study design,critical revision.
